# The Specific Mitogen- and Stress-Activated Protein Kinase MSK1 Inhibitor SB-747651A Modulates Chemokine-Induced Neutrophil Recruitment

**DOI:** 10.3390/ijms18102163

**Published:** 2017-10-17

**Authors:** Mokarram Hossain, Entesar Omran, Najia Xu, Lixin Liu

**Affiliations:** Department of Pharmacology, College of Medicine, University of Saskatchewan, Saskatoon, SK S7N 5E5, Canada; mokarram.hossain@ucalgary.ca (M.H.); eao266@mail.usask.ca (E.O.); nax537@campus.usask.ca (N.X.)

**Keywords:** MSK1, SB-747651A, neutrophil recruitment, intravital microscopy, chemokine

## Abstract

Mitogen-activated protein kinase (MAPK) signaling is involved in a variety of cellular functions. MAPK-dependent functions rely on phosphorylation of target proteins such as mitogen- and stress-activated protein kinase 1 (MSK1). MSK1 participates in the early gene expression and in the production of pro- and anti-inflammatory cytokines. However, the role of MSK1 in neutrophil recruitment remains elusive. Here, we show that chemokine macrophage inflammatory protein-2 (CXCL2) enhances neutrophil MSK1 expression. Using intravital microscopy and time-lapsed video analysis of cremasteric microvasculature in mice, we studied the effect of pharmacological suppression of MSK1 by SB-747651A on CXCL2-elicited neutrophil recruitment. SB-747651A treatment enhanced CXCL2-induced neutrophil adhesion while temporally attenuating neutrophil emigration. CXCL2-induced intraluminal crawling was reduced following SB-747651A treatment. Fluorescence-activated cell sorting analysis of integrin expression revealed that SB-747651A treatment attenuated neutrophil integrin α_M_β_2_ (Mac-1) expression following CXCL2 stimulation. Both the transmigration time and detachment time of neutrophils from the venule were increased following SB-747651A treatment. It also decreased the velocity of neutrophil migration in cremasteric tissue in CXCL2 chemotactic gradient. SB-747651A treatment enhanced the extravasation of neutrophils in mouse peritoneal cavity not at 1–2 h but at 3–4 h following CXCL2 stimulation. Collectively, our data suggest that inhibition of MSK1 by SB-747651A treatment affects CXCL2-induced neutrophil recruitment by modulating various steps of the recruitment cascade in vivo.

## 1. Introduction

During acute inflammation, neutrophils are recruited to the afflicted site by a well-defined and dynamic multi-step process that is regulated by a myriad of molecules and signaling cascades elicited by the cross-talk between neutrophils and endothelium [1,2]. The initial step of neutrophil rolling on the endothelium is followed by β_2_ integrin–ICAM-1-dependent adhesion of neutrophils to endothelium [3]. Adherent neutrophils then crawl in the vascular lumen to reach optimal emigration sites at endothelial junctions independently of hemodynamic forces, a process mediated by the α_M_β_2_ integrin Mac-1 [4]. Transendothelial migration of neutrophils is regulated by the interactions between integrins, PECAM-1 as well as junctional adhesion molecules and their respective ligands [1,2,3]. Neutrophil recruitment in vivo can be induced by CXC chemokines such as macrophage inflammatory protein-2 (CXCL2) [5]. Signaling mechanisms that regulate different steps of neutrophil recruitment such as intraluminal crawling and subsequent transendothelial migration of neutrophils are not completely understood.

Mitogen-activated protein kinases (MAPKs) are involved in a wide variety of cellular functions, such as differentiation, survival and apoptosis [6,7]. They are known to participate in the pathophysiology of neuronal and inflammatory diseases [7,8]. MAPK-dependent functions rely on phosphorylation of target proteins such as the closely related mitogen- and stress-activated protein kinases MSK1 and MSK2 [9]. Both kinases are phosphorylated by extracellular signal-regulated kinase ERK1/2 and by p38 MAPK and are, thus, activated by a wide range of physiological and pathological stimuli [9]. MSKs are homologous with the p90 ribosomal S6 kinase (RSK) family of kinases where the N-terminal kinase domains of both MSKs and RSKs are members of the AGC (protein kinase A, protein kinase G and protein kinase C) family of protein kinases [10]. Cellular functions of MSK1 include early gene expression [11] and apoptosis [12]. MSK1 regulates the activation of nuclear factor-κB (NF-κB) [13,14,15] and cyclic AMP response element-binding protein (CREB) [16], two transcription factors that are important in mediating inflammatory responses. MSK1 also regulates the production of pro- and anti-inflammatory cytokines [16,17,18,19,20] as well as endogenous mediators such as prostaglandin E_2_ [21]. However, the role of MSK1 in innate immunity remains elusive.

Cellular functions of MSK1 were previously elucidated by murine germline manipulation [20] and by inhibitors such as Ro 31-8220 and H89 [22,23]. However, these compounds are less selective and inhibit many other kinases [24]. Recently, SB-747651A was shown to be a highly selective and cell-active inhibitor of MSK1 with properties superior to H89 and Ro 31-8220 [24], thus enabling us to dissect the putative functions of MSK1 in vitro and in vivo.

In the present study, we explored the effect of pharmacological inhibition of MSK1 using SB-747651A on chemokine CXCL2-induced neutrophil recruitment in vivo. By using real-time intravital microscopy and time-lapsed video analysis, we simultaneously determined the multiple neutrophil recruitment parameters such as rolling, adhesion, emigration, intraluminal crawling velocity, transmigration time, detachment time, migration velocity, and chemotaxis index in tissue.

## 2. Results

**Figure 1:**
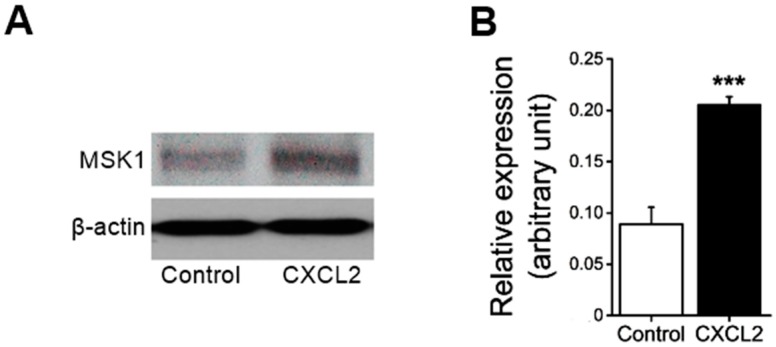
Effect of chemokine macrophage inflammatory protein-2 (CXCL2) on mitogen- and stress-activated protein kinase 1 (MSK1) expression in neutrophils. (**A**) Representative original Western blot and (**B**) means ± SEM (*n* = 4) showing total MSK1 expression determined in 1-h saline-treated (Control) or CXCL2-treated (30 nM at 37 °C for 1 h) bone marrow neutrophils (relative to β-actin). *** (*p* < 0.001) from the Control.

First, we examined whether the treatment of neutrophils with CXC chemokine CXCL2 affects MSK1 protein expression. As shown in Figure 1, treatment of mouse bone marrow neutrophils with CXCL2 significantly enhanced MSK1 protein expression in neutrophils. 

To test whether CXCL2-sensitive MSK1 participates in neutrophil recruitment in vivo, we studied the effect of the specific MSK1 inhibitor SB-747651A on neutrophil-endothelial cell interactions using intravital microscopy of post-capillary venule in mouse cremaster muscle. To this end, superfusion of murine cremaster muscle with SB-747651A (5 µM) for 30 min prior to and for 1 h following the placement of CXCL2-containing gel significantly enhanced leukocyte rolling flux (83.7 ± 3.4 cells/min with SB-747651A treatment versus 48.3 ± 3.1 cells/min without SB-747651A treatment; *n* = 4, *p* < 0.01) and rolling velocity (64.4 ± 2.8 µm/s with SB-747651A treatment versus 46.9 ± 5.8 µm/s without SB-747651A treatment; *n* = 4, *p* < 0.05) in comparison to CXCL2-treated group in the absence of SB-747651A treatment. 

**Figure 2:**
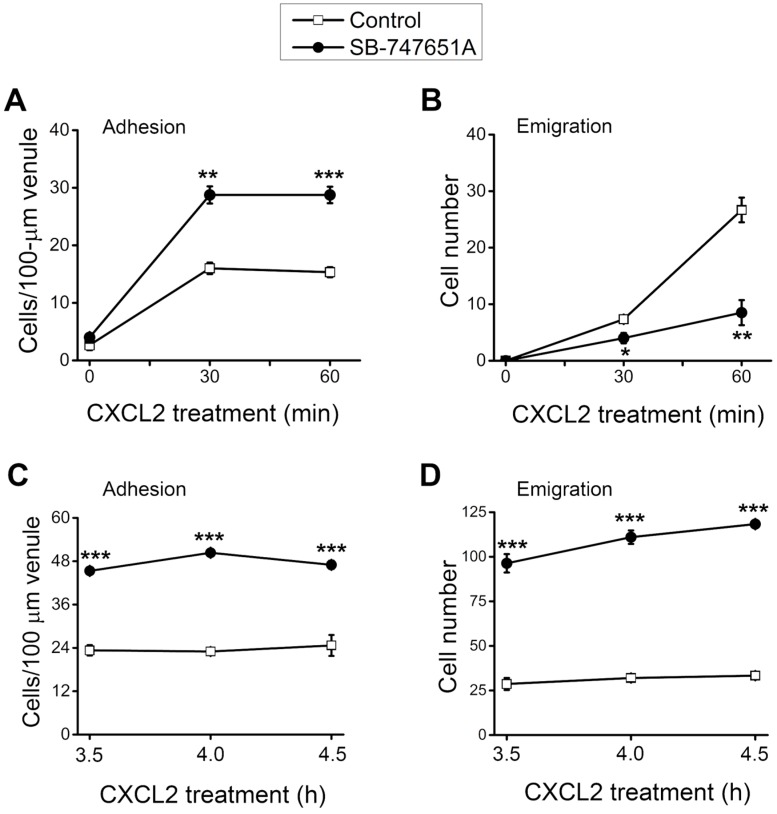

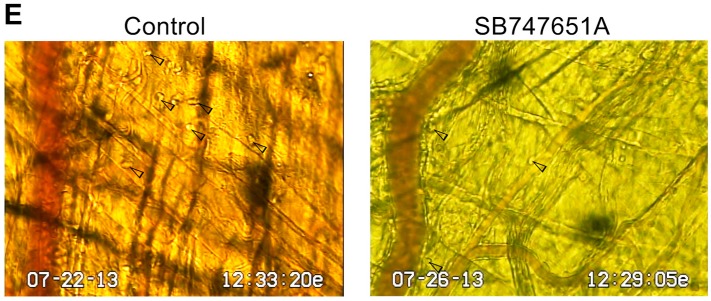
Effect of SB-747651A on CXCL2-induced neutrophil adhesion and emigration. (**A**) Time course of the number of adherent neutrophils (cells/100-μm venule) and (**B**) time course of the number of emigrated neutrophils (cells/235 × 208 μm^2^ field) induced by CXCL2 in the absence (Control) or in the presence of MSK1 inhibitor SB-747651A (5 µM) 30 min prior to and 60 min following the placement of CXCL2-containing gel. Data are means ± SEM (*n* = 4). *, ** and *** indicate significant difference (*p* < 0.05, *p* < 0.01 and *p* < 0.001, respectively) from the Control. (**C**) Time course of the number of adherent neutrophils (cells/100-μm venule) and (**D**) time course of the number of emigrated neutrophils (cells/443 × 286 μm^2^ field) induced by CXCL2 in the absence (Control) or in the presence of MSK1 inhibitor SB-747651A (3 mg/kg, intrascrotal injection, 1-h prior to CXCL2 treatment) at 3.5–4.5 h following an intrascrotal injection of 0.2 µg CXCL2. Data are means ± SEM (*n* = 3). *** indicates significant difference (*p* < 0.001) from the Control. (**E**) Representative images from intravital video microscopy showing a postcapillary venule (**Left**) and the surrounding cremaster muscle with emigrated neutrophils (arrow head) at 60 min induced by CXCL2 in the absence (Control) or in the presence of MSK1 inhibitor SB-747651A (5 µM) 30 min prior to and 60 min following the placement of CXCL2-containing gel (**Right**).

During neutrophil recruitment, not all rolling neutrophils became adherent and emigrated in the microvasculature. To analyze the subsequent steps of neutrophil recruitment in the same cremaster muscle, we visualized the neutrophil recruitment process and determined the number of adherent and emigrated neutrophils. As depicted in Figure 2A, the number of adherent neutrophils was significantly increased at 30–60 min after the placement of CXCL2-containing gel on the cremaster muscle, an effect significantly more pronounced in the presence of SB-747651A treatment. Similarly, the number of emigrated neutrophil was significantly increased at 30–60 min after placement of CXCL2-containing gel on the cremaster muscle, an effect that was significantly reduced by the SB-747651A treatment (Figure 2B). Additional experiments were conducted to explore the consequence of SB-747651A treatment on prolonged stimulation with CXCL2. To this end, SB-747651A treatment (3 mg/kg intrascrotal injection, 1 h prior to the administration of CXCL2) resulted in increased neutrophil adhesion 3.5–4.5 h following stimulation with CXCL2 (0.2 µg intrascrotal injection) as compared to the effect of CXCL2 stimulation alone (Figure 2C). The number of emigrated neutrophils was significantly increased after SB-747651A treatment at 3.5–4.5 h following CXCL2 stimulation as compared to the CXCL2 treatment alone (Figure 2D).

**Figure 3:**
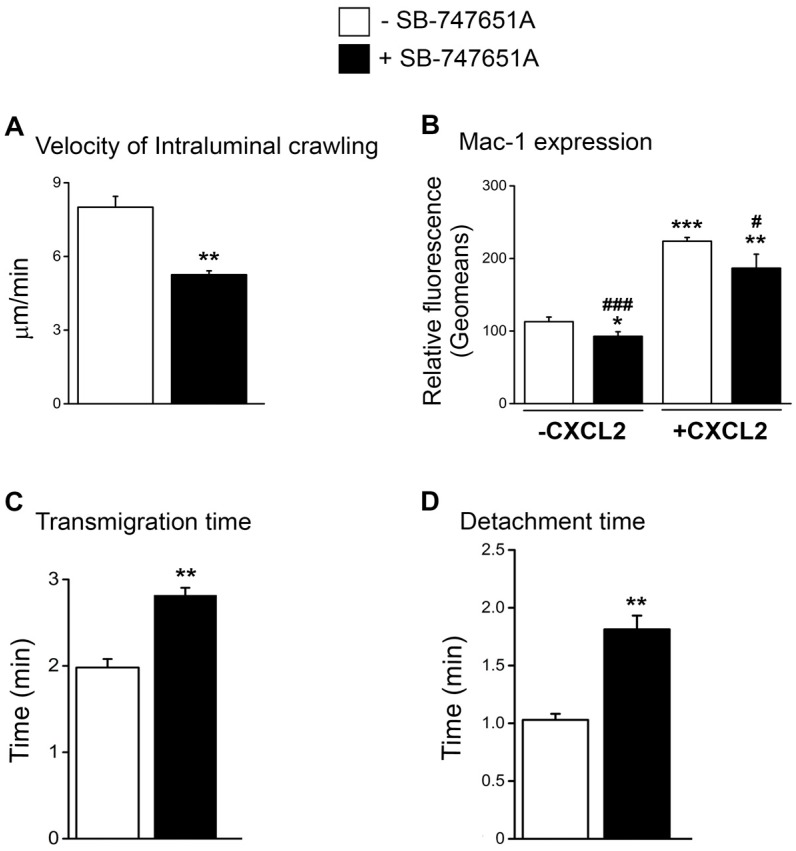
Effect of SB-747651A on CXCL2-induced Mac-1-dependent intraluminal crawling and transendothelial migration. (**A**) The velocity of intraluminal crawling (µm/min) of neutrophils crawling in the luminal surface of the endothelium upon stimulation with CXCL2 in the absence (Control, white bar) or in the presence (black bar) of MSK1 inhibitor SB-747651A (5 µM) 30 min prior to and 60 min following the placement of CXCL2-containing gel. Data are means ± SEM (*n* = 4). ** indicates significant difference (*p* < 0.01) from the Control. (**B**) Means ± SEM (*n* = 3) of Mac-1-dependent fluorescence expressed as geomeans in untreated neutrophils (Control; white bar) and in neutrophils treated with SB-747651A (5 µM, 30 min prior to addition of CXCL2; black bar) in the absence (**left** bars) or in the presence (**right** bars) of stimulation with CXCL2 (30 nM for 10 min). *, **, and *** indicate significant difference (*p* < 0.05, *p* < 0.01, and *p* < 0.001, respectively) from the Control without CXCL2. ^#^ and ^###^ indicate significant difference (*p* < 0.05 and *p* < 0.001, respectively) from the group without SB-747651A. (**C**) The duration (min) of neutrophil transmigration across the endothelium and (**D**) the detachment time (min) of neutrophils from the venule upon stimulation with CXCL2 in the absence (Control, white bar) or in the presence (black bar) of MSK1 inhibitor SB-747651A (5 µM) 30 min prior to and 60 min following the placement of CXCL2-containing gel. Data are means ± SEM (*n* = 4). ** indicates significant difference (*p* < 0.01) from the Control.

Reduced emigration of neutrophils following treatment with SB-747651A at early time points following CXCL2 stimulation could be due to the impairment in the early neutrophil recruitment steps subsequent to adhesion. To explore the effect of SB-747651A treatment on neutrophil intraluminal crawling and transendothelial migration, we analyzed neutrophil intraluminal crawling using time-lapsed video photography. As shown in Figure 3A, the velocity of intraluminal crawling in response to CXCL2 chemotactic gradient was significantly lower following SB-747651A treatment as compared to the CXCL2 control. These data suggest that SB-747651A treatment thwarts the intraluminal crawling of adherent neutrophils to optimal sites of emigration. Intraluminal crawling of adherent neutrophils is dictated by neutrophil α_M_β_2_ integrin Mac-1 [4]. Flow cytometry analysis revealed that neutrophil Mac-1 expression was significantly increased following the treatment of bone marrow neutrophils with CXCL2, an effect significantly blunted by SB-747651A treatment (Figure 3B and Figure S1). We also determined the effect of SB-747651A on the expression of integrin α_L_β_2_, LFA-1, another important β_2_ integrin on CXCL2-treated neutrophils and found that CXCL2 only marginally enhanced LFA-1 expression on neutrophils and SB-747651A was completely ineffective on the LFA-1 expression level in the presence or absence of CXCL2 (Figure S2). These results suggest that SB-747651A treatment affects CXCL2-induced intraluminal crawling of neutrophils in a Mac-1-dependent manner.

To further define the cause of the reduced early emigration following SB-747651A treatment, we analyzed transmigration time and detachment time of neutrophils in response to CXCL2 chemotactic gradient. As a result, SB-747651A treatment significantly increased transmigration time and detachment time as compared to the control without this inhibitor (Figure 3C,D) indicating a slower process of neutrophil emigration. These data suggest that SB-747651A treatment affects mechanisms that regulate transendothelial migration of neutrophils in response to CXCL2 chemotactic gradient.

**Figure 4:**
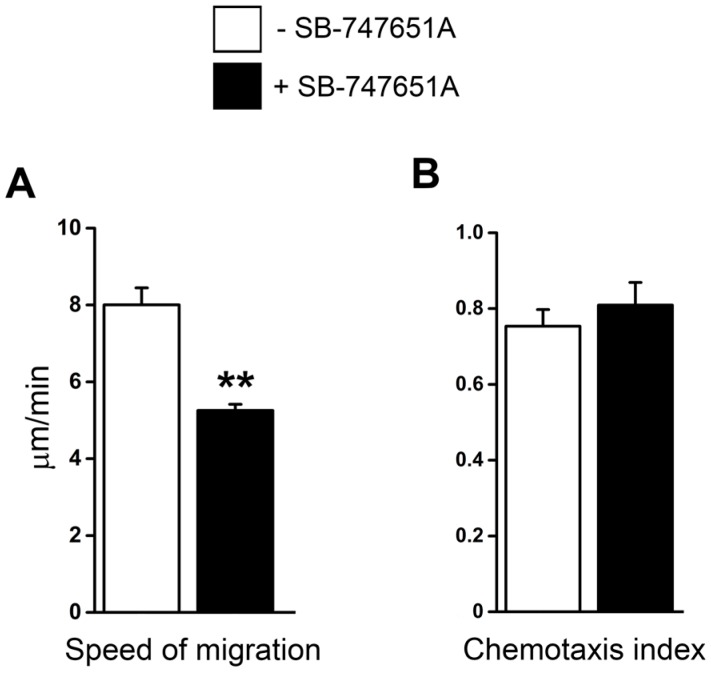
Effect of SB-747651A on CXCL2-induced neutrophil migration and chemotaxis in tissue. (**A**) The neutrophil migration speed (µm/min) and (**B**) the chemotaxis index of migrating neutrophils in response to CXCL2 stimulation in the absence (Control, white bar) or in the presence (black bar) of MSK1 inhibitor SB-747651A (5 µM) 30 min prior to and 60 min following the placement of CXCL2-containing gel. Data are means ± SEM (*n* = 4). ** indicates significant difference (*p* < 0.01) from the Control.

Next, we performed an additional series of experiments to elucidate whether SB-747651A treatment modulates extravascular migration of neutrophils in tissue. Time-lapsed video microscopy analysis revealed that in response to CXCL2 chemotactic gradient, the speed of neutrophil migration was significantly reduced following SB-747651A treatment compared to the CXCL2 control group (Figure 4A). Chemotaxis index, a parameter of migration directionality, measures the ratio of the distance in the direction toward CXCL2-gel to the total migration distance the cell moved in the tissue. In response to the CXCL2 chemotactic gradient, chemotaxis index of migrating neutrophils was, however, not altered following SB-747651A treatment as compared to the CXCL2 control (Figure 4B). These data indicate that SB-747651A treatment inhibits the migration speed of extravascular chemotaxing neutrophils but does not affect their directionality in response to CXCL2 chemotactic gradient.

**Figure 5:**
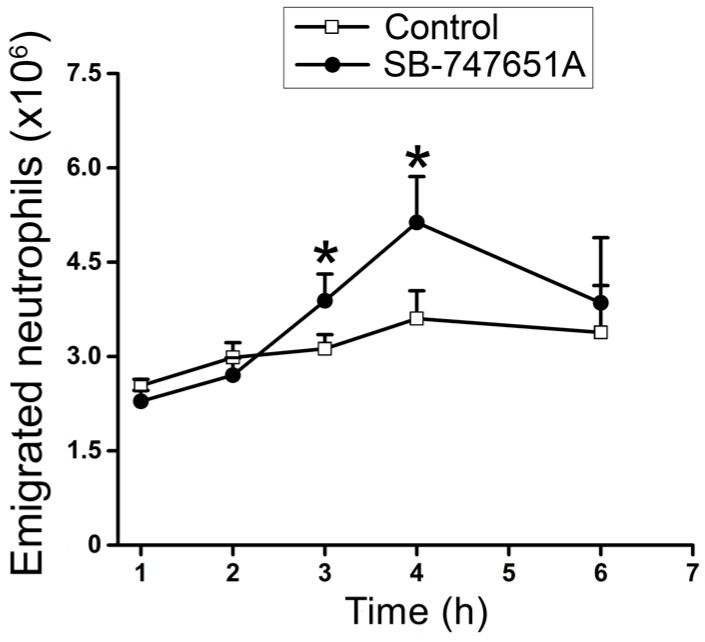
Effect of SB-747651A on CXCL2-induced neutrophil emigration in peritoneum. Time course of the number of emigrated neutrophils (×10^6^ cells) counted in the peritoneal lavage fluid in the absence (Control, open square) or in the presence of MSK1 inhibitor SB-747651A (3 mg/kg, i.p. 30 min prior to CXCL2 injection, solid circle) collected at 1, 2, 3, 4, and 6 h after an i.p. injection of CXCL2 (0.5 µg/mouse). Data are means ± SEM (*n* = 3). * indicates significant difference (*p* < 0.05) from the Control.

To corroborate the effects of SB-747651A treatment on CXCL2-induced transendothelial migration of neutrophils in vivo, an additional series of experiments were performed to explore the effect of SB-747651A treatment on CXCL2-triggered infiltration of neutrophils to the peritoneal cavity. As shown in Figure 5, SB-747651A treatment did not enhance neutrophil emigration at 1–2 h after CXCL2 treatment but significantly increased the number of emigrated neutrophils in the peritoneal lavage fluid following 3 and 4 h of CXCL2 injection, indicating that SB-747651A treatment affects neutrophil extravasation by increasing neutrophil emigration only at 3 and 4 h in mouse peritonitis model of acute inflammation.

## 3. Discussion

Neutrophil-endothelial cell interactions during acute inflammation generate molecular signals that are decisive in the recruitment of neutrophils to the site of inflammation. The present study discloses the effect of pharmacological inhibition of MSK1 on different steps of chemokine CXCL2-induced neutrophil recruitment. We show that in response to chemokine CXCL2, MSK1 protein expression was upregulated in neutrophils. Pharmacological inhibition of MSK1 by using selective MSK1 inhibitor SB-747651A enhanced CXCL2-induced adhesion of neutrophils to the microvascular lumen while temporarily curtailing transendothelial migration of neutrophils. SB-747651A treatment thwarted Mac-1-dependent intraluminal crawling, while increasing both transmigration time and detachment time, effects favoring reduced transendothelial migration. SB-747651A treatment further mitigated the migration speed of neutrophils in extravascular tissue. 

Mechanistically, MSK1 targets both pro- and anti-inflammatory genes [17]. Molecules upstream of MSK1 signaling, ERK1/2 and p38 MAPK, are important in the production of inflammatory cytokines [17]. These signaling molecules also activate negative feedback pathways via MSK1/2 to suppress the proinflammatory effects of Toll-like receptor 4 (TLR4) signaling [20]. Mice deficient in MSK1/2 were shown to be more susceptible to endotoxic shock and showed enhanced myeloperoxidase activity following phorbol ester-triggered eczema [20]. Similarly, skin inflammation was shown to be enhanced in MSK1/2-deficient mice with elevated infiltration of neutrophils in response to oxazolone-induced allergic contact dermatitis [25]. Furthermore, MSK1/2 activation was also shown to be involved in the pathogenesis of psoriatic skin lesions [26]. Discordantly, however, suppression of MSK1 by inhibitors such as H89 showed amelioration of airway inflammation [27]. In another study, MSK1 is documented to participate in airway inflammation elicited by respiratory syncytial virus [19]. Discrepancies in the effect of MSK1 inhibitors and the anti-inflammatory phenotype of MSK1-deficient mice may well be explained by the non-specificity of the inhibitors Ro 31-8220 and H89 to different cellular kinases reported in the earlier studies. 

The role of MSK1 in neutrophil-endothelial cell interactions remains elusive. In addition to neutrophils, endothelial cells also express MSK1, which participates in the activation of CREB [28] and in the regulation of synthesis of platelet-activating factor [29]. In the present study, however, the role of endothelial MSK1 in the observed effects of SB-747651A treatment on CXCL2-induced neutrophil recruitment cannot be ruled out. Thus, further investigations are warranted to examine the role of cell-specific regulation of neutrophil recruitment by MSK1 using MSK1 knockout mice.

While p38 MAPK was previously shown to regulate neutrophil adhesion and transendothelial migration [30], more recent work has suggested that p38 MAPK also contributes to other steps of neutrophil recruitment, such as Mac-1-dependent intraluminal crawling and extravascular migration [5]. Intracellular signals regulating neutrophil intraluminal crawling involve Vav guanine nucleotide exchange factor 1 (Vav1) and mammalian-actin binding protein 1 downstream of spleen tyrosine kinase [31,32]. However, the role of MSK1 in neutrophil intravascular crawling and extravascular chemotaxis was not investigated in the previous studies. Enhanced adhesion has been expected to be translated into increased transendothelial migration. Transendothelial migration is effectively accomplished by subsequent recruitment steps following neutrophil adhesion and intraluminal crawling. We observed that SB-747651A treatment attenuated CXCL2-stimulated Mac-1 expression and CXCL2-induced intraluminal crawling of neutrophils. Surprisingly, despite increased neutrophil adhesion to the vascular endothelium, we found that SB-747651A treatment effectively decreased intraluminal crawling and decreased transendothelial migration at the early time points, the latter was evidenced by the decreased emigration, prolonged transmigration time and detachment time of neutrophils at early time points during transendothelial migration following SB-747651A treatment. In contrast to the role of p38 MAPK on the directionality of chemotaxing neutrophils [5], inhibition of MSK1 did not affect the chemotaxis index of extravascular migrating neutrophils but attenuated the migration speed of neutrophils. SB-747651A treatment presumably affects the expression of adhesion molecules that regulate the passage of neutrophils into extravascular tissue. It is intriguing to speculate that differential effects of SB-747651A treatment on neutrophil and endothelial adhesion molecules may have accounted for the increased adhesion and decreased emigration of neutrophils during the very early stage of recruitment in acute inflammation. It is also possible that the attenuation of the migration speed of neutrophils in tissue by SB-747651A increases the accumulation of emigrated neutrophils in the inflammatory sites at time points later than 1–2 h after CXCL2 stimulation. It is interesting to note that SB-747651A did not change neutrophil emigration in early time points but increased neutrophil emigration until 3–4 h in peritoneum stimulated by CXCL2. This suggests that SB-747651A treatment may only result in enhanced neutrophil recruitment in peritoneum after 3–4 h of CXCL2 treatment, in a pattern different from the two-phase recruitment in cremaster muscle.

Collectively, our data suggest that inhibition of MSK1 by SB-747651A treatment affects CXCL2-induced neutrophil recruitment by modulating various steps of the recruitment cascade in vivo.

## 4. Materials and Methods

### 4.1. Mice

Male C57BL/6N mice between 8- and 16-weeks-old, purchased from Charles River Canada (Saint-Constant, QC, Canada), were used in experiments. This study was carried out with the approved animal protocols (#20070028; 28 November 2012 and 7 June 2013) from the University Committee on Animal Care and Supply (UCACS) at the University of Saskatchewan following the standards of Canadian Association of Animal Care. 

### 4.2. Intravital Microscopy

Mice were anaesthetized with an intraperitoneal (i.p.) injection of 10 mg/kg xyalzine (Bayer, Toronto, ON, Canada) and 200 mg/kg ketamine hydrochloride (Rogar, Montreal, QC, Canada). The mouse cremaster muscle preparation was used to study neutrophil behaviour in microcirculation and tissue as described previously [5,33,34,35]. The cremaster muscle was kept warm and superfused with 37 °C-warmed bicarbonate-buffered saline (pH 7.4; containing in mM 133.9 NaCl, 4.7 KCl, 1.2 MgSO_4_ and 20 NaHCO_3_). An upright microscope (model Eclipse Ci-s, Nikon, Tokyo, Japan) with a LUCPLFLN 20× objective lens was projected to a charge-coupled device (CCD) color video camera (DC-220, Dage, Dage-MTI, Inc., Michigan City, IN, USA) for bright-field intravital microscopy. For the induction of neutrophil recruitment, two approaches were taken. In the first approach, an agarose gel at 1-mm^3^ size containing murine CXC chemokine CXCL2 (0.5 µM; R&D Systems, Minneapolis, MN, USA) was placed on the surface of the cremaster muscle in a preselected area 350-μm distant from and parallel to the observed postcapillary venule. After placing a glass coverslip to hold the gel, the cremaster muscle was superfused with bicarbonate-buffered saline at a very slow rate (≤10 μL/min) to allow the formation of CXCL2 chemotactic gradient. Throughout the experiment, neutrophil behaviour and hemodynamic changes in the selected cremasteric postcapillary venule (25–40 μm diameter) were visualized on a TV monitor and recorded at real time on a DVD recorder before (for time 0 min) and after the addition of CXCL2-containing gel (recorded for 60 min). During recording, all efforts were made to adjust and keep the microscope images focused on the adhering, crawling, transmigrating and chemotaxing neutrophil inside the venule and in the muscle tissue. The number of rolling, adherent, and emigrated neutrophils was determined in the cremasteric microvasculature during offline playback analysis of the recorded video as described previously [34]. Where indicated, the specific MSK1 inhibitor 5 µM SB-747651A (Axon Medchem BV, Groningen, The Netherlands) was superfused on the cremaster muscle 30 min prior to and remained superfused for 60 min after the addition of CXCL2-containing gel. The second approach was to induce neutrophil recruitment at later time points by intrascrotal injection of CXCL2 (0.2 µg in 100 µL sterile saline) and by determining the parameters of neutrophil recruitment under intravital microscopy at 3.5–4.5 h after CXCL2 treatment. In this approach, where indicated, SB-747651A was administered at 3 mg/kg by intrascrotal injection 1 h prior to CXCL2 injection.

### 4.3. Cell Tracking

Using ImageJ software (Version 1.48, National Institutes of Health, Bethesda, MD, USA), neutrophil intraluminal crawling, transmigration, and chemotaxis in cremasteric microvasculature were analyzed using the time-lapsed movie converted from the real-time video recording of the experiment as described previously [4,5,35,36]. The following recruitment parameters were quantified from tracking and analyzing at least 40 cells for each treatment group: (a) velocity of intraluminal crawling (µm/min): the total distance the neutrophil crawled from the initial site of adhesion to the transmigration site (µm) divided by the duration of neutrophils undergoing intraluminal crawling (min); (b) transmigration time (min): from the time the neutrophil stopped crawling and started to transmigrate to the time the whole neutrophil body was just outside the venule; (c) detachment time (min): from the time the neutrophil body was just outside the venule after its transmigration to the time when the neutrophil migrated away and lost contact to the venule; (d) speed of migration in tissue (µm/min): neutrophil migration distance in tissue (µm) divided by the time that the neutrophil migrated (min); (e) chemotaxis index in tissue: the ratio of the distance in the direction toward the CXCL2-gel to the total migration distance the neutrophil moved in tissue.

### 4.4. Isolation of Murine Neutrophils

Bone marrow cells were freshly harvested from mouse femurs and tibias, and the marrow was flushed with ice-cold Ca^2+^- and Mg^2+^-free phosphate-buffered saline (PBS) solution. Neutrophils were isolated using a Percoll (GE Healthcare, Uppsala, Sweden) gradient (72%, 64%, and 52%) centrifugation at 1060× *g* at room temperature for 30 min as described previously [37] and subsequently washed with PBS. The isolated cells had >85% purity of morphologically mature neutrophils.

### 4.5. Fluorescence-Activated Cell Sorting (FACS) Analysis of Mac-1 and LFA-1 Expression

The expression of β_2_ integrins Mac-1 and LFA-1 on neutrophils was determined using a previously described method with slight modifications [5,38]. Following lysis of red blood cells, bone marrow-derived neutrophils were incubated at 37 °C for 30 min in the presence or absence of 5 µM SB-747651A in vitro. The cells were stimulated with CXCL2 (30 nM at 37 °C for 10 min) to upregulate Mac-1 and LFA-1 expression. Aliquots of the neutrophil suspension (10^6^/mL) were washed in ice-cold PBS containing 1% BSA, stained with a fluorescent anti-Mac-1 or anti-LFA-1 antibody (Anti-mouse CD11b FITC; clone M1/70; anti-mouse CD11a FITC; clone M17/4, both from eBioscience, San Diego, CA, USA) or the isotype control (Rat IgG2bκ FITC; eBioscience) and incubated for 30 min at 4 °C. The samples were then centrifuged (1200 rpm, 3 min, 4 °C) and washed twice with ice-cold PBS containing 1% BSA and analyzed in the FL-1 channel of an Epics XL flow cytometer (Beckman Coulter, Miami, FL, USA) with an excitation wavelength of 488 nm and an emission wavelength of 530 nm. 

### 4.6. Induced Peritonitis

Acute mouse peritonitis was induced to obtain emigrated neutrophils after an i.p. injection of murine CXCL2 (0.5 µg in sterile saline). Cells were then lavaged and harvested from the peritoneum at different time points and the emigrated neutrophils were counted. 

### 4.7. Western Blotting

After the indicated treatment, bone marrow neutrophils were lysed in lysis buffer (pH 8.0; containing 50 mM Tris–HCl, 150 mM NaCl, 1% NP-40, 0.5% sodium deoxycholate, 0.1% SDS and protease and phosphatase inhibitor cocktails, purchased from Fisher Scientific, Toronto, ON, Canada). Proteins (40 µg) were solubilized in Laemmli sample buffer at 95°C for 5 min and resolved by 10% SDS–PAGE. For immunoblotting, proteins were transferred onto a nitrocellulose membrane and blocked with 5% BSA in Tris-buffered saline-Tween 20 at room temperature for 1 h. Then, the membrane was incubated with anti-MSK1 antibody (1:1000; Cell Signaling Technology, Danvers, MA, USA) at 4 °C overnight. After incubation with horseradish peroxidase-conjugated goat anti-rabbit secondary antibody (1:2000; Santa Cruz Biotechnology, Santa Cruz, CA, USA) for 1 h at room temperature, antibody binding was detected with the ECL detection reagent (GE Healtcare, Baie d’Urfe, QC, Canada). β-actin (mouse anti-β-actin antibody, 1:1000, Santa Cruz Biotechnology) was detected after stripping with a buffer (pH 6.8; containing 0.5 M Tris–HCl, 2% SDS and 0.7% 2-β-mercaptoethanol). Densitometric quantification of the detected bands was performed using Gene Snap Software (Syngene, Frederick, MD, USA).

### 4.8. Statistical Analysis

Data are expressed as means ± SEM. *n* denotes the number of mice studied in each group or the number of mice used to derive bone marrow neutrophils for in vitro studies. Statistical analysis was performed using two-tailed Student’s *t*-test and *p* values < 0.05 were considered statistically significant.

## Supplementary Materials

Supplementary materials can be found at www.mdpi.com/1422-0067/18/10/2163/s1.
