# Supplementary material for: The Specific Mitogen- and Stress-Activated Protein Kinase MSK1 Inhibitor SB-747651A Modulates Chemokine-Induced Neutrophil Recruitment
Source: Int J Mol Sci. 2017 Oct 17;18(10):2163. doi: 10.3390/ijms18102163 (PMC5666844; doi:10.3390/ijms18102163)
Supplement: Supplementary file 1 [file ijms-18-02163-s001.pdf]

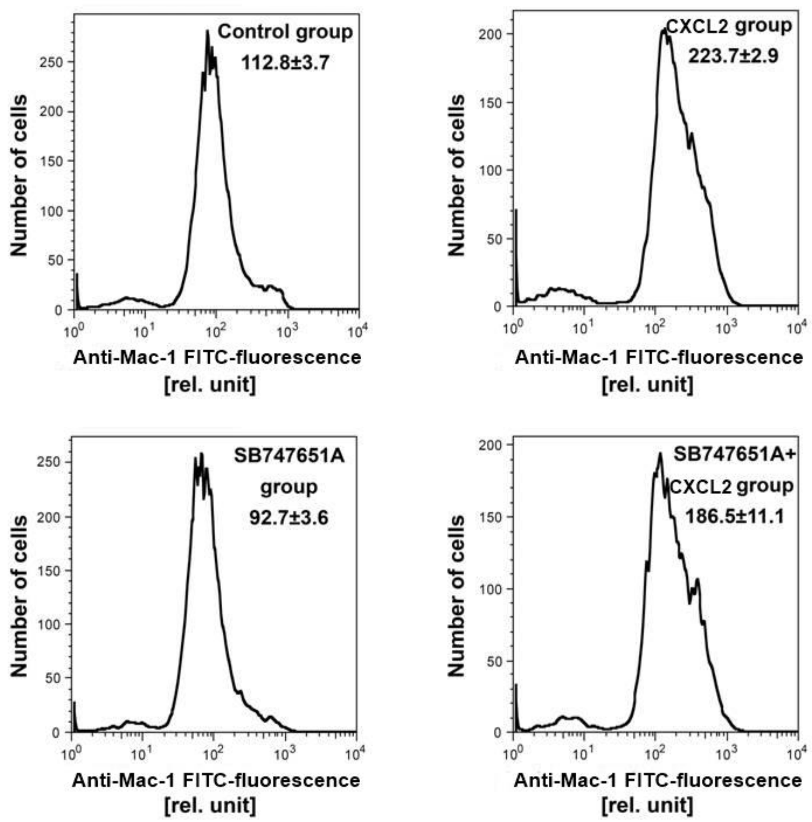

Supplemental Figure S1. Original histogram of Mac-1 dependent fluorescence in bone marrow-derived neutrophils with or without SB747651A treatment and in the presence or absence of CXCL2 (both treatments are described in the Material and Methods section).

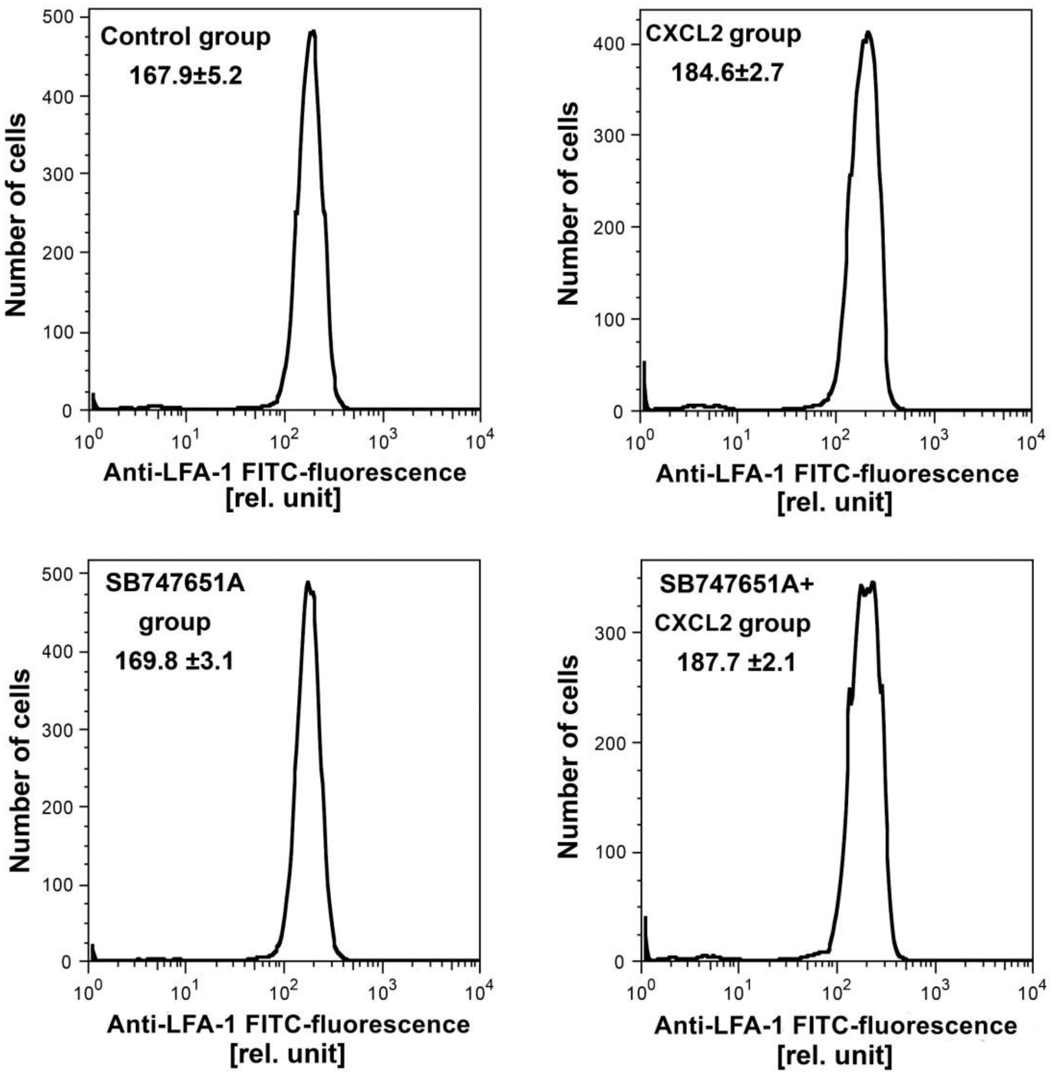

Supplemental Figure S2. Original histogram of LFA-1 dependent fluorescence in bone marrow-derived neutrophils with or without SB747651A treatment and in the presence or absence of CXCL2 (both treatments are described in the Material and Methods section).
